# Recent and rapid anthropogenic habitat fragmentation increases extinction risk for freshwater biodiversity

**DOI:** 10.1111/eva.13128

**Published:** 2020-09-18

**Authors:** Chris J. Brauer, Luciano B. Beheregaray

**Affiliations:** ^1^ Molecular Ecology Laboratory, College of Science and Engineering Flinders University Adelaide SA Australia

**Keywords:** conservation genomics, eco‐evolutionary dynamics, genetic rescue, Murray–Darling Basin, riverine barriers, riverscape genomics, teleost fish, threatened biodiversity

## Abstract

Anthropogenic habitat fragmentation is often implicated as driving the current global extinction crisis, particularly in freshwater ecosystems. The genetic signal of recent population isolation can be confounded by the complex spatial arrangement of dendritic river systems. Consequently, many populations may presently be managed separately based on an incorrect assumption that they have evolved in isolation. Integrating landscape genomics data with models of connectivity that account for landscape structure, we show that the cumulative effects of multiple in‐stream barriers have contributed to the recent decline of a freshwater fish from the Murray–Darling Basin, Australia. In addition, individual‐based eco‐evolutionary simulations further demonstrate that contemporary inferences about population isolation are consistent with the 160‐year time frame since construction of in‐stream barriers began in the region. Our findings suggest that the impact of very recent fragmentation may be often underestimated for freshwater biodiversity. We argue that proactive conservation measures to reconnect many riverine populations are urgently needed.

## 1 INTRODUCTION

We are now confronted by the sixth global mass extinction with the current rate of species losses far exceeding pre‐anthropogenic background estimates (Barnosky et al., 2011). This crisis is particularly severe in freshwater ecosystems, which have shown declines of biodiversity greater than for either terrestrial or marine ecosystems (Darwall et al., 2018). Habitat loss and fragmentation are key factors leading to the genetic and demographic decline of populations that together threaten species persistence (Fischer & Lindenmayer, 2007). Over the last century, close to one million large dams and many millions of smaller in‐stream barriers have been constructed globally (Jackson et al., 2001; Liermann et al., 2012). These barriers have had devastating ecological consequences by preventing or restricting connectivity among populations, leading to higher rates of genetic drift and inbreeding. This, in turn, can lead to lower fitness due to inbreeding depression and reduced evolutionary potential due to loss of genetic diversity (Frankham, 2005; Keyghobadi, 2007). Additionally, small populations become more vulnerable to extirpation due to stochastic demographic events (Lande, 1993) and, when this occurs on a regional scale, species extinctions are the inevitable result (Hanski, 1998).

Landscape genetics provides a way to identify how human activities threaten the persistence of wild populations (Manel & Holderegger, 2013). The time lag between environmental change and any detectable genetic signal resulting from this change can, however, make it very difficult to disentangle the effects of historical from contemporary processes (Landguth et al., 2010). This is particularly the case for naturally structured populations such as those found in dendritic river networks (Coleman et al., 2018). The progression from landscape genetics to landscape genomics has increased both the spatial and temporal resolutions at which evolutionary processes can be examined, offering a more powerful framework with which to quantify the effects of very recent disturbance on populations (Allendorf et al., 2010; Grummer et al., 2019). Previous landscape genetics studies investigating the impact of in‐stream barriers have often focused on larger, migratory species or assessed only one, or a few large barriers (Faulks et al., 2011; Gouskov et al., 2016; Meeuwig et al., 2010; Mims et al., 2019; Torterotot et al., 2014). For example, Muhlfeld et al. (2012) used microsatellite loci and simulations to understand the impact of placement of a single barrier on introgressive hybridization between native westslope cutthroat trout (*Oncorhynchus clarkii lewisi*) and non‐native rainbow trout in Glacier National Park, USA. On the other hand, small‐bodied but ecologically important species often receive relatively little attention from conservation managers (Olden et al., 2007; Saddlier et al., 2013). Regional‐scale efforts to improve fish passage in Australia have been successful in restoring passage along the main river channel for large‐bodied species (Barrett & Mallen‐Cooper, 2006; Baumgartner et al., 2014); however, these measures have proved ineffective for most small fishes (Harris et al., 2017). The cumulative impact of numerous smaller in‐stream barriers (e.g., weirs, farm dams and road crossings) is likely to greatly impact small‐bodied and nonmigratory fishes; however, this has been the subject of much less research at a regional scale (Coleman et al., 2018; Diebel et al., 2015; but see Nathan et al., 2019).

In this landscape genomics study, we examine the effects of recent habitat fragmentation on the southern pygmy perch (*Nannoperca australis*), a threatened small‐bodied fish (<80 mm) that recently experienced major demographic declines and local extinctions across the Murray–Darling Basin (MDB), Australia (Brauer et al., 2016; Cole et al., 2016; Hammer et al., 2013). This ecological specialist is restricted to small streams and wetlands, is typical of many native small‐bodied fishes in the region and offers a conservative model for guiding broader conservation strategies as the impacts of fragmentation are likely to be more pronounced for larger, migratory species. Since European colonization, freshwater habitat in the MDB has rapidly deteriorated due to severe water overharvesting, land clearing, habitat loss and fragmentation (Davies et al., 2010; Kingsford, 2000), and the MDB is now considered one of Australia’s most vulnerable and threatened ecosystems (Laurance et al., 2011). The MDB has very few natural in‐stream barriers, but it has been heavily modified with more than 10,000 dams, weirs, road crossings, levees and barrages constructed since the late 1850s (Baumgartner et al., 2014). As such, the MDB provides a unique opportunity to examine the consequences of recent habitat fragmentation without the confounding influence of prolonged human disturbance over hundreds of years as is common to many northern hemisphere river basins (e.g., Hansen et al., 2014). Environmental factors, including human disturbance, are known to influence genetic diversity for *N. australis* (Brauer et al., 2016; Cole et al., 2016); however, little is known about the specific role that widespread habitat fragmentation has played in the species recent and rapid decline. We hypothesize that, after accounting for historical patterns of genetic structure, genetic differentiation among demes should increase with the number of in‐stream barriers separating them. We also predict that populations most isolated by fragmentation would exhibit reduced effective population size (*N*_e_) and lower levels of genetic diversity. Additionally, we used forward genetic simulations to investigate whether high contemporary levels of genetic differentiation could have arisen in the relatively short time since the construction of in‐stream barriers began in the MDB. Our results demonstrate that recent anthropogenic habitat fragmentation has contributed to the loss of genetic diversity and population isolation observed. They also suggest that proactive conservation measures to restore connectivity (e.g., environmental flows, habitat restoration) and increase evolutionary potential (e.g., genetic rescue) are urgently required for this, and potentially many other poorly dispersing aquatic species.

## 2 METHODS

### 2.1 Sampling, ddRAD genotyping and SNP filtering

**FIGURE 1:**
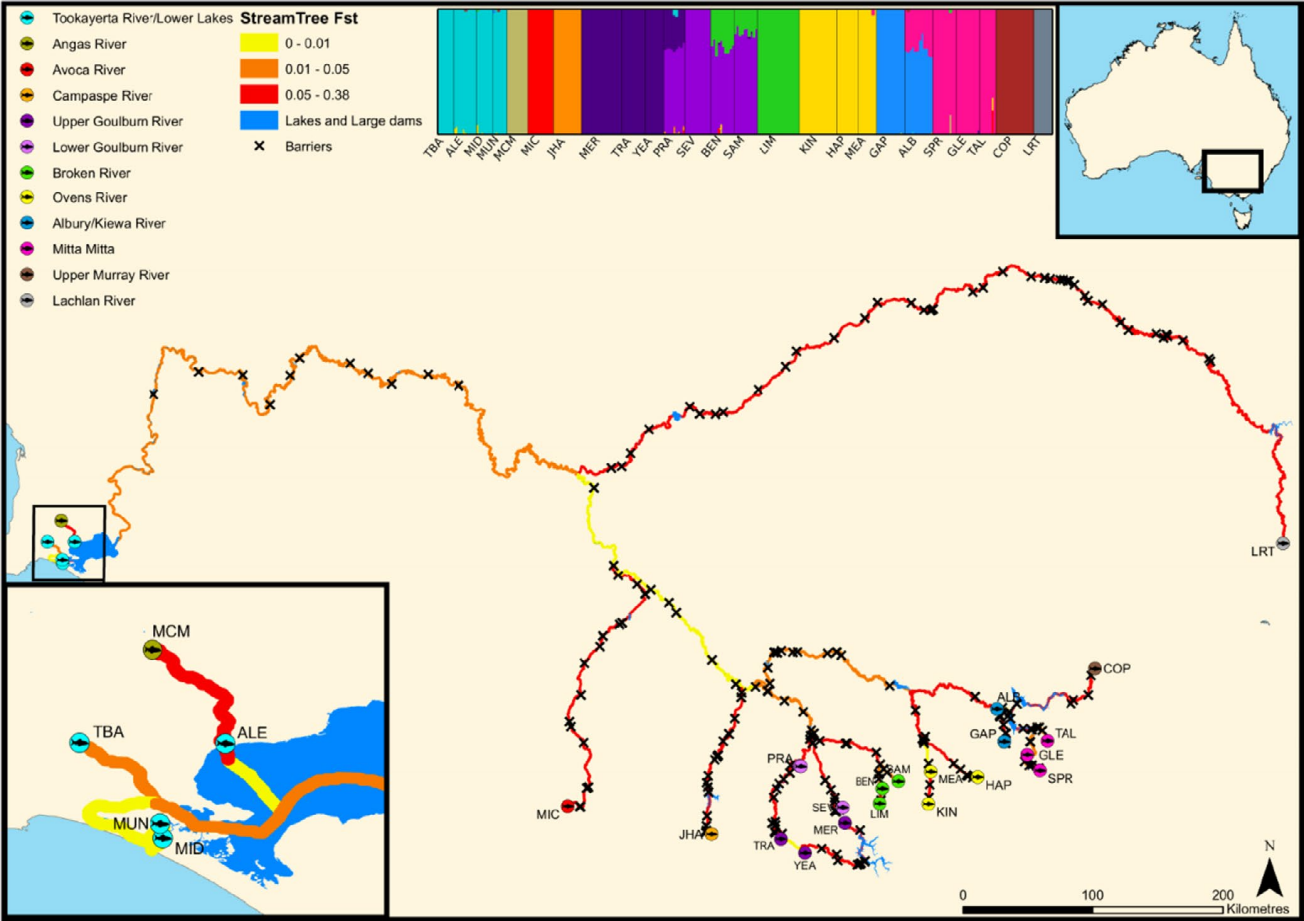
*Nannoperca australis* sampling locations in the Murray–Darling Basin (MDB). Stream sections are colour coded according to *F*_ST_ estimated using the *StreamTree* model (Kalinowski et al., 2008). Cross markers represent the location of artificial in‐stream barriers. Admixture plot is based on 3,443 SNPs depicting K = 12 clusters determined by maximum marginal likelihood using fastStructure (Raj et al., 2014)

A total of 263 individuals were sampled from 25 locations, encompassing 13 catchments across the entire current MDB distribution of *N. australis* between 2000 and 2013 (Figure 1; Table 1). Samples were obtained from a single sampling event for all except three populations (MER, LIM and KIN; Table 1) for which samples were obtained in both 2009 and 2013 to increase the number of samples. To minimize the number of cohorts sampled per population, we targeted adult fish of similar size from each sampling site. To avoid the inclusion of highly related individuals in the data, we estimated pairwise relatedness among individuals from each site using the dyadic likelihood relatedness estimator described in Milligan (2003) and implemented in the R package related (Pew et al., 2015). Fish were ethically euthanized using clove oil, frozen in liquid nitrogen in the field and stored at −70°C in the Australian Biological Tissues Collection at the South Australian Museum, Adelaide. Collections were obtained under permits from various state fisheries agencies, and research was performed in accordance with Flinders University Animal Welfare Committee policies and under approval E313.

**TABLE 1:** Sample size (N), expected heterozygosity (*H*_E_), population‐specific *F*_ST_ (Weir & Hill, 2002) and effective population size estimates (*N*_e_)

Catchment	Site	*N*	*H*_E_	*F*_ST_	*N*_e_ (95% CI)
**Tookayerta (TOO)**	**TBA**	**7**	**0.227**	**0.059**	**∞**
**Lower Lakes (LMR)**	**ALE**	**10**	**0.263**	**0.066**	**198.6 (158.6–264.9)**
	**MID**	**7**	**0.262**	**0.092**	190.9 (163.3–229.4)*
	**MUN**	**6**	**0.260**	**0.034**	
Angas (ANG)	MCM	9	0.097	0.555	76.3 (61.0–101.3)
Avoca (AVO)	MIC	11	0.114	0.409	13.7 (13.2–14.4)
Campaspe (CAM)	JHA	12	0.091	0.364	393.8 (184.0–∞)
Upper Goulburn (UGO)	MER	17	0.075	0.467	70.4 (61.4–82.2)
	TRA	10	0.075	0.433	50.7 (41.2–65.3)
	YEA	8	0.087	0.364	260.4 (111.1–∞)
Lower Goulburn (LGO)	PRA	9	0.243	0.179	114.9 (98.4–137.9)
	SEV	11	0.218	0.119	54.8 (50.8–59.4)
Broken (BRO)	BEN	10	0.236	0.159	117.2 (101.7–138.2)
	SAM	10	0.234	0.188	124.7 (108.0–147.2)
	LIM	18	0.118	0.337	99.1 (88.5–112.5)
Ovens (OVE)	KIN	16	0.104	0.297	69.9 (62.1–79.8)
	HAP	9	0.114	0.369	∞
	MEA	8	0.158	0.245	53.4 (45.7–64)
Kiewa (KIE)	GAP	12	0.168	0.305	122.5 (105.3–146.2)
Albury (ALB)	ALB	12	0.226	0.299	305.4 (241.8–413.4)
Mitta Mitta (MIT)	SPR	10	0.152	0.262	98.1 (80.5–125)
	GLE	10	0.143	0.408	51.1 (46.1–57.2)
	TAL	7	0.164	0.479	31.9 (29.1–35.2)
Upper Murray (COP)	COP	16	0.133	0.297	118.7 (102.2–141.1)
Lachlan (LAC)	LRT	8	0.057	0.672	18.1 (15.3–21.8)

Note

Lowland wetland sites referred to as Lower Murray in the text are indicated in bold. *MID and MUN samples combined for *N*_e_ estimation.

DNA was extracted using the Qiagen DNeasy Blood and Tissue Kit according to the manufacturers protocol. DNA integrity and purity were assessed using gel electrophoresis and a NanoDrop 1000 spectrophotometer (Thermo Scientific), respectively. Sequencing libraries were prepared in‐house based on a double‐digest restriction site‐associated DNA (ddRAD) library protocol (Peterson et al., 2012). Samples were multiplexed with 48 samples per Illumina HiSeq2000 lane and sequenced as paired‐end, 100‐bp reads. Raw sequences were demultiplexed using the *process_radtags* module of *Stacks* v.1.04 (Catchen et al., 2011) before using dDocent v.1.2 (Puritz et al., 2014) for de novo reference catalogue assembly and genotyping. The data were then filtered to retain only variants present in at least 70% of individuals and in 70% of populations, retaining only one biallelic SNP per locus with a minimum minor allele frequency of 0.05.

Population structure and other demographic parameters such as effective population size should be assessed using neutral loci (Allendorf et al., 2010; Luikart et al., 2003). To define a putatively neutral data set, *F*_ST_ outlier loci were detected using a Bayesian approach with BayeScan v.2.1 (Foll & Gaggiotti, 2008) and the coalescent‐based FDIST method (Beaumont & Nichols, 1996) in Arlequin v.3.5 (Excoffier & Lischer, 2010). BayeScan was run for 100,000 iterations using prior odds of 10,000. Loci different from zero with a *q*‐value < 0.1 were considered outliers. Arlequin was run specifying the hierarchical island model with 50,000 simulations of 100 demes for each of 13 populations (based on the 13 separate catchments sampled). Loci outside the neutral distribution at a false discovery rate (FDR) of 10% were considered outliers. Loci detected as outliers by either BayeScan or Arlequin were filtered. The remaining SNPs were examined for departure from expectations of Hardy–Weinberg equilibrium (HWE) using GenoDive 2.0b27 (Meirmans & Van Tienderen, 2004). Finally, loci out of HWE at a FDR of 10% in more than 50% of populations were removed. Detailed information concerning library preparation and bioinformatics are described in Appendix S1.

### 2.2 Population structure

Pairwise *F*_ST_ (Weir & Cockerham, 1984) was estimated among sampling sites using GenoDive (Meirmans & Van Tienderen, 2004) with significance assessed using 10,000 permutations. Bayesian clustering analysis of individual genotypes was then performed using fastStructure (Raj et al., 2014). Ten independent runs for each value of *K* (1–25) were completed to ensure consistency, and the most likely *K* was assessed by comparing the model complexity that maximized marginal likelihood across replicate runs.

### 2.3 Anthropogenic isolation of populations

If anthropogenic habitat fragmentation has affected population connectivity and dispersal, we should expect genetic differentiation to increase in response to the number of in‐stream barriers separating populations. To determine whether local characteristics of the stream network (i.e., in‐stream barriers and other local‐scale landscape heterogeneity) better explain population differentiation than isolation by distance (IBD), we used the StreamTree model of Kalinowski et al. (2008). Genetic distances among populations were modelled as the sum of all pairwise genetic distances that mapped to each section of the stream network. This provides a distance measure that is independent of the length of each stream section and identifies the reaches that contribute most to restricting gene flow (e.g., due to dendritic structure, in‐stream barriers or other local landscape effects). Model fit was assessed by plotting the StreamTree fitted distance against observed *F*_ST_ and calculating the regression coefficient of determination (*R* ^2^). This model was then compared with a model of IBD calculated using multiple matrix regression with randomization (MMRR) following the method of Wang (2013). Pairwise population distances along the river network were calculated with ArcMap v.10.2 (ESRI, 2012). Model significance for the MMRR was assessed using 10,000 random permutations.

In dendritic river systems, hierarchical network structure and spatial hydroclimatic variation can also drive patterns of genetic diversity of stream‐dwelling organisms (Fourcade et al., 2013; Hughes et al., 2009; Morrissey & de Kerckhove, 2009; Thomaz et al., 2016). To evaluate the relative contributions of anthropogenic habitat fragmentation, natural stream hierarchy and environmental variation, we again used MMRR. In addition to IBD, we used distance matrices calculated for the number of in‐stream barriers, catchment membership and a range of environmental variables. The number of in‐stream barriers separating sites was determined using spatial data from the Murray–Darling Basin Weir Information System (Murray–Darling Basin Authority, 2013). To account for the effect of dendritic stream hierarchy, a binary model matrix describing catchment membership was constructed such that pairwise comparisons of sites from within the same catchment were assigned a value of zero, and comparisons among catchments were scored as one. Finally, a subset of 40 hydroclimatic variables was obtained from the Australian hydrological geospatial fabric (Geoscience Australia, 2011; Stein et al., 2014). These were assigned to one of five categories describing variation in temperature, precipitation, flow regime, human disturbance and topography. Variance inflation factor (VIF) analysis was then used to exclude highly correlated variables using a VIF threshold of 10 (Dyer et al., 2010). The remaining variables were reduced to principal components (PCs) using the dudi.pca function in the ADE4 R package (Dray et al., 2016), and Euclidean distance matrices were constructed based on the PCs with eigenvalues > 1 (Yeomans & Golder, 1982) retained for each category. All distance matrices were z‐transformed to facilitate direct comparison of partial regression coefficients (Schielzeth, 2010). Each variable was initially tested in an independent univariate MMRR before significant factors were combined in a multivariate MMRR model with 10,000 random permutations used to assess significance.

### 2.4 Habitat fragmentation, genetic diversity and population size

To test the hypothesis that the most isolated populations exhibit reduced genetic diversity, we examined the relationship between population‐specific *F*_ST_ and expected heterozygosity (*H*_E_). Population‐specific *F*_ST_ was estimated for each sampling site using the method of Weir and Hill (2002), and *H*_E_ was calculated using Genodive.

Effective population size was estimated using the linkage disequilibrium (LD) estimator implemented in NeEstimator 2.01 (Do et al., 2014). This method is based on the assumption that LD at independently segregating loci in a finite population is a function of genetic drift and performs particularly well with a large number of loci and where population sizes are expected to be small (Waples & Do, 2010). In the absence of significant *F*_ST_, Lower Murray sites MID and MUN were considered one population and these samples were combined for the *N*_e_ estimates. NeEstimator was run assuming random mating and using a *P*_crit_ value of 0.075 following guidelines for small sample sizes suggested by Waples and Do (2010).

### 2.5 Eco‐evolutionary simulations

Simulation studies are becoming an increasingly important part of landscape genomics as a wide range of parameters can be explored for key evolutionary processes such as gene flow, genetic drift, mutation and selection (Hoban et al., 2012). In this case, we used simulations to examine whether levels of contemporary population isolation are consistent with having evolved during the time since barrier construction began in the MDB. Additionally, the simulations were designed to assess the additive effects of simultaneously increasing the amount of fragmentation and reducing the habitat patch size of each deme (i.e., multiple in‐stream barriers fragmenting populations into smaller and smaller patches, that are able to support smaller and smaller populations). We simulated three metapopulation sizes (*N*_e_ = 1,000, *N*_e_ = 500 and *N*_e_ = 100) using SLiM 3.1 (Haller & Messer, 2018). Each simulation was based on a 1D stepping stone population model assuming equal *N*_e_ for each subpopulation while maintaining a constant metapopulation size to simulate a concurrent increase in the number of barriers and reduction in habitat patch size. Each simulation consisted of four 100 Kb genomic elements and assumed a constant mutation rate of 10^−7^ and recombination rate of 10^−8^. Each simulation was first run for a burn‐in phase of 20,000 generations with a migration rate of 0.5 between adjacent subpopulations to generate diversity and allow the system to reach migration–drift equilibrium with *F*_ST_=~0. Although this almost certainly underestimates historical population structure before anthropogenic disturbance, this figure provides a conservative approach by maximizing the number of generations required to evolve current levels of differentiation. Following the burn‐in, the construction of barriers was simulated by setting the migration rate among demes to zero for 300 generations. Nine models with an increasing number of demes (2–10) were simulated for each metapopulation size to examine the effect of increasing levels of fragmentation (Figure S1‐S3), and 100 replicate runs of each scenario were completed. The ‐weir‐fst‐pop command of VCFtools (Danecek et al., 2011) was used to calculate *F*_ST_ for each replicate. To estimate the time required to reach current levels of observed population differentiation, assuming a generation time of one year (Humphries, 1995), the number of generations (mean of the 100 replicates) needed to achieve *F*_ST_ = 0.2 (mean contemporary *F*_ST_ within upper Murray catchments = 0.196) was plotted against the number of fragments for each scenario for the three metapopulation models. Scripts used to perform the simulations and analyses are available at https://github.com/pygmyperch/SPP_SLiM.

## 3 RESULTS

### 3.1 Sampling, ddRAD genotyping and SNP filtering

Following demultiplexing, 1,602,903,910 forward and reverse sequencing reads were recovered. A total of 2,589,251 variant sites were genotyped with dDocent, and after filtering, 5,162 high‐quality SNPs were retained. We removed 873 unique *F*_ST_ outlier loci identified by BayeScan and Arlequin, along with a further 846 loci found to be outside HWE expectations in > 50% of populations. Following the relatedness analysis, all individuals were retained as no highly related pairs were present, and just five pairs of potential half‐sibs were identified from the 1,377 pairwise comparisons. This resulted in a final, putatively neutral data set of 3,443 SNPs for the 263 individuals (Table S1).

### 3.2 Population structure

High levels of population genetic structure were evident between most demes of *N. australis,* with pairwise comparisons of *F*_ST_ among sampling sites ranging from 0–0.79 (global *F*_ST_ = 0.48). All pairwise *F*_ST_ estimates were significant (*p* < 0.003) except between immediately adjacent lower MDB sites MID and MUN (*F*_ST_= −0.002, *p* = 0.66) (Table S2). Results from fastStructure indicated population boundaries are strongly correlated with natural riverine catchment structure, with *K* = 12 identified as the most likely number of populations (Figure 1). This is consistent with a previous microsatellite study based on a larger sample (578 individuals; 45 localities) that inferred that, until the recent European colonization in the MDB, well‐connected metapopulations of *N. australis* existed within its catchments (Cole et al., 2016).

### 3.3 Anthropogenic isolation of populations

**FIGURE 2:**
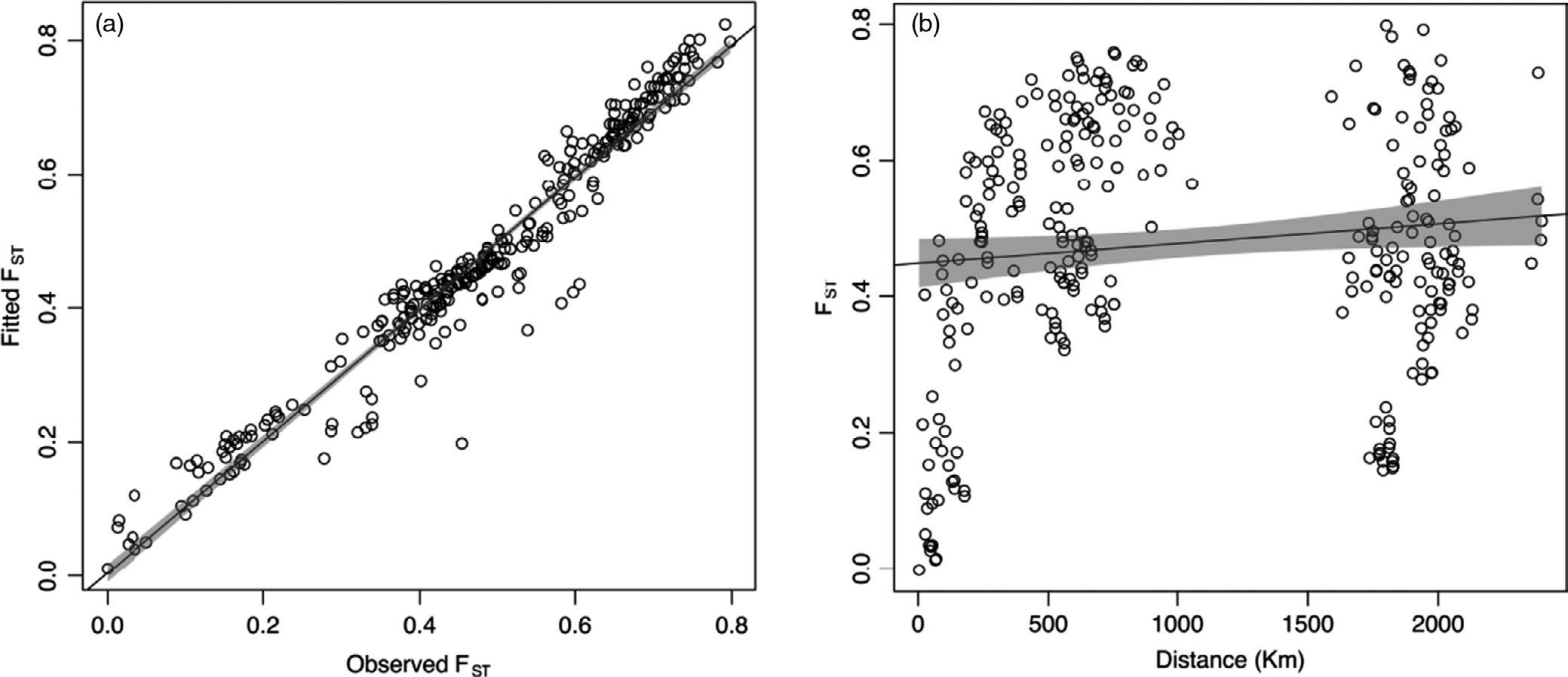
Plots of (a) *StreamTree* analyses and b) isolation by distance (IBD) for *Nannoperca australis* in the MDB. The *StreamTree* plot compares fitted *F*_ST_ based on the *StreamTree* model with observed pairwise *F*_ST_ values (*R* ^2^ = 0.947, *β* = 0.986 [0.959–1.012 95% CI], *p* < 2 × 10^−16^). The IBD plot depicts the relationship between pairwise *F*_ST_ and riverine distance between sampling sites (*R* ^2^ = 0.0139, *β* = 0.108 [0.004–0.212 95% CI], *p* = 0.343). Shaded area represents the 95% confidence interval

The StreamTree model was used to identify parts of the stream network that contribute more to *F*_ST_ (e.g., restricted dispersal due to barriers or other local environmental conditions). Results indicated that local characteristics of the stream network better explain *F*_ST_ than the null hypothesis of IBD (i.e., the resistance to dispersal for any given stream section is determined by its length). Figure 1 provides a visual representation of the relationship between StreamTree fitted distance and the density of artificial in‐stream barriers, with stream sections colour coded according to *F*_ST_ as estimated by the model (yellow represents a modelled local *F*_ST_ range of 0−0.01, orange: 0.01−0.05 and red: 0.05−0.38) and the location of barriers marked with **X**. The StreamTree model was a good fit for the data and was significantly related to observed *F*_ST_ (*R* ^2^ = 0.947, *β* = 0.986 [0.959−1.012 95% CI], *p* < 2 × 10^‐16^) (Figure 2a), whereas IBD was not significant (*R* ^2^ = 0.0139, *β* = 0.108 [0.004−0.212 95% CI], *p* = 0.343) (Figure 2b). Although there was significant IBD within‐catchment groups (i.e. the first cluster in Figure 2b, *R* ^2^ = 0.730, *β* = 0.0016 [0.001–0.002 95% CI], *p* = 6.54 × 10^−8^), IBD was not significant in models across the whole basin, in contrast to models of stream hierarchy and barriers (see below). In addition, even when comparisons were limited to sites within catchments, the number of barriers still provided a better model than IBD (*R* ^2^ = 0.81 versus 0.73, respectively; Figure S4).

Following VIF analyses, 19 environmental variables from the five categories were retained. The first two PCs for temperature, flow and topographic variables scored eigenvalues > 1, while only one component each for the precipitation and human disturbance PCAs scored an eigenvalue > 1, so individual variables rather than PCs for these categories were retained. This resulted in a final list of six hydroclimatic PCs and five individual precipitation and disturbance variables (Table S3).

**FIGURE 3:**
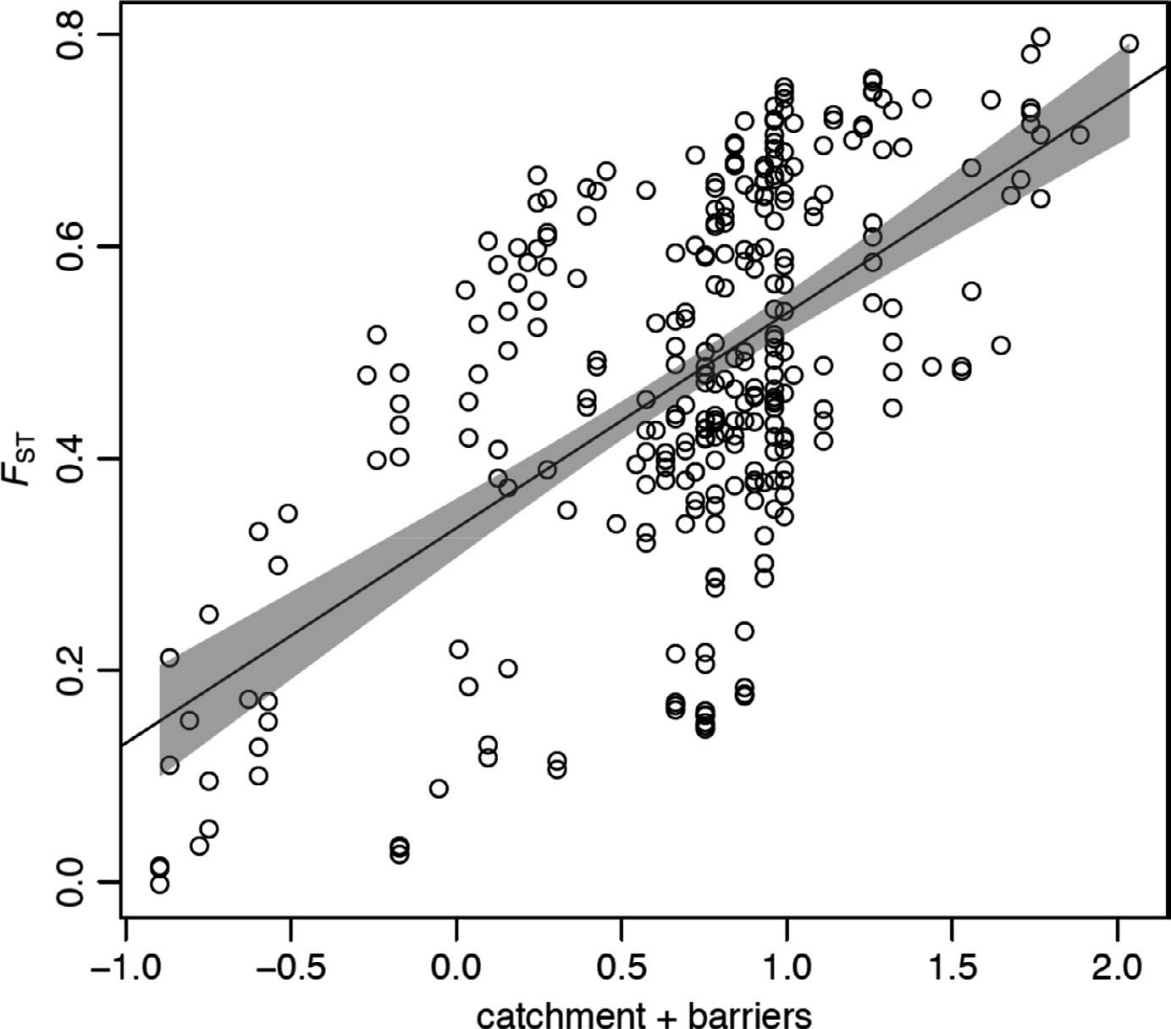
Multiple matrix regression with randomization (MMRR) plot for the combined effects of natural stream hierarchy (model matrix of catchment membership) and number of barriers on *F*_ST_ (*R* ^2^ = 0.358, *β*_catchment_ = 0.725 [0.374–1.076 95% CI], *β*_barriers_ = 0.462 [0.365–0.560 95% CI], *p* < 0.0001). Shaded area represents the 95% confidence interval

Assessment of the relative influence of anthropogenic habitat fragmentation, natural stream hierarchy and environmental heterogeneity indicated that population structure is driven by a combination of the effects of stream network hierarchy and the number of in‐stream barriers. Univariate regressions revealed catchment membership (*R* ^2^ = 0.170, *β* = 0.449 [0.336–0.562 95% CI], *p* < 0.0001) and the number of in‐stream barriers separating sites (*R* ^2^ = 0.322, *β* = 0.548 [0.458–0.639 95% CI], *p* < 0.0001) were both good predictors of population differentiation, while there was no evidence for isolation by environment (Table 2). Including both significant predictors (catchment membership and number of barriers) in a multivariate model improved model fit with catchment membership, and the number of barriers each accounting for 61% and 39% of the explained variation, respectively (*R* ^2^ = 0.358, *β*_catchment_ = 0.725 [0.374–1.076 95% CI], *β*_barriers_ = 0.462 [0.365–0.560 95% CI], *p* < 0.0001) (Figure 3 and Table 2).

**TABLE 2:** Results of multiple matrix regression with randomization (MMRR) tests for the relationship between pairwise genetic distance (*F*_ST_) and geographic distance, catchment membership, number of in‐stream barriers and environmental distances

Model	Variable	Coefficient		95% CI	*p*‐value	*R* ^2^	Model *p*‐value
	Distance	0.108	0.004– 0.213		0.3340	0.014	
	Catchment	0.449	0.336– 0.562		**0.0001**	0.170	
	Barriers	0.548	0.458– 0.639		**0.0001**	0.322	
	TempPC1	−0.130	−0.233‐ −0.028		0.2465	0.021	
	TempPC2	0.180	0.077– 0.282		0.1443	0.039	
	CATCOLDQRAIN	0.098	−0.007– 0.202		0.3813	0.011	
	CATDRYQRAIN	−0.061	−0.170– 0.043		0.5515	0.004	
	STRWETQRAIN	−0.058	−0.162– 0.046		0.5496	0.004	
	FlowPC1	−0.053	−0.158– 0.051		0.6698	0.003	
	FlowPC2	−0.125	−0.227‐ −0.023		0.3520	0.019	
	CDI	0.037	−0.068– 0.142		0.6571	0.002	
	FRDI	−0.087	−0.190– 0.015		0.4603	0.009	
	TopoPC1	−0.121	−0.225‐ −0.017		0.2368	0.017	
	TopoPC2	0.021	−0.083– 0.125		0.8644	0.001	
Catchment + Barriers						0.358	**0.0001**
	Catchment	0.725	0.374– 1.076		**0.0045**		
	Barriers	0.462	0.365– 0.560		**0.0001**		

Note

Pairwise environmental distances between each site were calculated as Euclidean distance for each environmental variable and principal component (PC) described in Brauer et al. (2016). *p*‐values < 0.0001 are indicated in bold.

### 3.4 Habitat fragmentation, genetic diversity and population size

Genetic diversity varied across the MDB with an average *H_E_* of 0.161 (0.057–0.263). There was a sharp contrast between regions with average *H_E_* of 0.253 for sites in the more connected Lower Murray wetlands, compared to 0.143 for sites in the highly fragmented upper reaches (Table 1). A strong negative relationship between population‐specific *F*_ST_ and *H_E_* was also evident (*R* ^2^ = 0.737, *β* = −2.05 [−2.58‒−1.52 95% CI], *p* < 1 × 10^−7^) with the most isolated populations also harbouring the least genetic variation (Figure S5; Table 1). Effective population size estimates were generally low, averaging 194.75 for Lower Murray sites and 112.26 for sites in the upper reaches, with many of the latter < 100 (Table 1).

### 3.5 Eco‐evolutionary simulations

**FIGURE 4:**
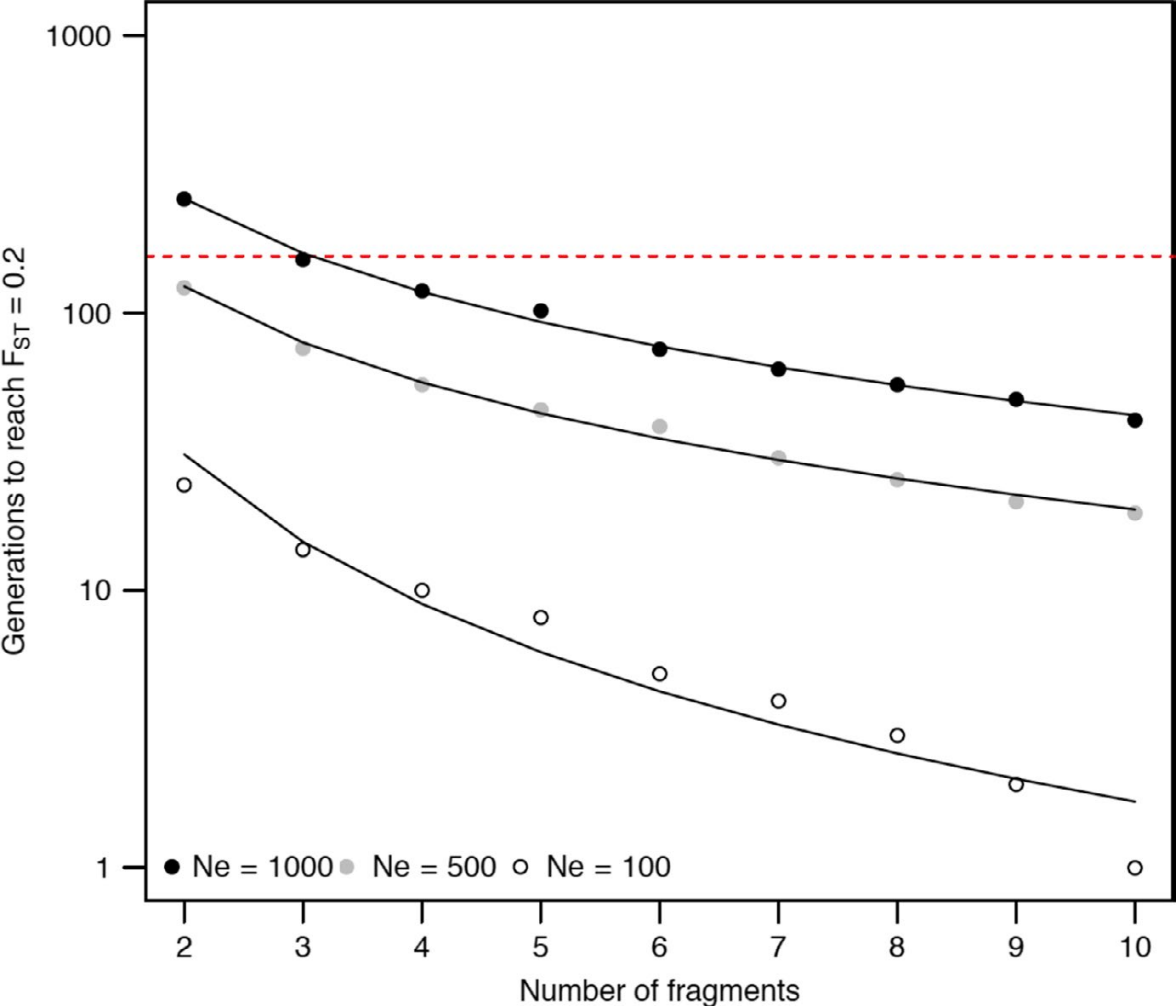
Number of generations (log scale) for global *F*_ST_ to reach 0.2 with increasing levels of habitat fragmentation for simulated *N. australis* metapopulations of *N*_e_ = 1,000, *N*_e_ = 500 and *N*_e_ = 100. Simulations were based on a stepping stone model assuming equal *N*_e_ for each subpopulation and were allowed to run for 20,000 generations with a migration rate of 0.5 between adjacent demes before 300 generations with no migration. Red dashed line indicates the approximate number of generations since construction of in‐stream barriers began in the MDB (160 generations)

The simulations demonstrated that contemporary population differentiation among sites within catchments (mean within headwater catchments *F*_ST_ = 0.196) could have evolved from a more connected system within the time since the construction of in‐stream barriers began ~160 generations ago (Figure 4; Table S4; Appendix S3‐S5). For metapopulations with an *N*_e_ of 1,000, *F*_ST_ approached 0.2 in less than 160 generations with only three barriers fragmenting the population. Models assuming *N*_e_ = 500 and *N*_e_ = 100 indicated that substantially fewer generations following fragmentation were required to reach contemporary levels of *F*_ST_. At *N*_e_ = 500, *F*_ST_ = 0.2 occurred after 124 generations with one barrier and after just 19 generations with nine barriers (Figure 4; Table S4). For smaller populations of *N*_e_ = 100, contemporary levels of differentiation evolved within 24 generations with just one barrier (Figure 4; Table S4).

## 4 DISCUSSION

Habitat fragmentation is a key process implicated in the current and unprecedented worldwide loss of freshwater biodiversity (Fischer & Lindenmayer, 2007). Determining the contribution of recent human activities to the decline of riverine species is, however, challenging, as the genetic signal of recent disturbance can be confounded by historical patterns of dispersal shaped by hydrological network structure (Brauer et al., 2018; Coleman et al., 2018; Landguth et al., 2010). Integrating landscape genomics data with models of connectivity that account for landscape structure, we show that the cumulative effects of multiple in‐stream barriers have contributed to the recent decline of a freshwater fish from the Murray–Darling Basin, Australia. Populations most isolated by recent habitat fragmentation exhibited reduced genetic diversity and increased population differentiation, and this signal remained strong after accounting for the historical effects of dendritic stream hierarchy.

Interestingly, we found no evidence for isolation by environment (IBE), despite a previous genotype–environment association (GEA) study for the same species finding several hydroclimatic variables influenced putatively adaptive genetic variation at both regional and local scales (Brauer et al., 2016). This is likely due to the ability of GEA methods to identify signal from relatively few regions of the genome responding to selection (Forester et al., 2018). In contrast, tests for IBE based on *F*_ST_ are expected to be more sensitive to the impact of genetic drift in shaping patterns of genome‐wide variation (De Mita et al., 2013). In this case, the strong effect of drift due to habitat fragmentation isolating populations and reducing population sizes has likely eroded any *F*_ST_‐based IBE signal for *N. australis* that may have existed in the past.

Results from the simulations suggested that over a 160 generation time period, just a single in‐stream barrier is sufficient to generate contemporary levels of population differentiation (average within‐catchment *F*_ST_ = 0.2) among previously homogeneous sites for populations up to *N*_e_ = 500, and just two barriers are required for *N*_e_ = 1,000. These findings are consistent with the hypothesis that anthropogenic habitat fragmentation has contributed to isolation of populations since European colonization of the region. Our estimates of local *N*_e_ sizes for *N. australis* were low, averaging approximately 200 for Lower Murray sites and just over 100 for sites in the upper reaches. While single sample *N*_e_ estimates should be interpreted cautiously (Waples et al., 2014), the approach we used is known to perform well for small populations (Do et al., 2014; Waples & Do, 2010), and our results are consistent with expectations based on remnant habitat patch sizes, and estimates obtained in an earlier microsatellite study (Cole et al., 2016). Other previous work based on coalescent analyses of microsatellite DNA data sets has demonstrated that historical population sizes of *N. australis* were much larger before European colonization (Attard et al., 2016), and that populations across the MDB were also more connected until that time (Cole et al., 2016). Together, our findings support these studies and the hypothesis that the low genetic diversity, small *N*_e_ and high *F*_ST_ observed for contemporary populations likely reflects the combined impact of both historical and recent processes, rather than being due solely to natural demographic variability over longer evolutionary time scales. In addition, several populations sampled for this study have subsequently suffered local extirpation during prolonged drought, and the small size of most remnant populations indicate they are at high risk of extinction.

Since the 1800s, land use and hydrology in the MDB has been increasingly modified due to urbanization and irrigation (Leblanc et al., 2012). These changes have included the construction of thousands of barriers to fish passage across the basin (Baumgartner et al., 2014), and it is now considered one of Australia’s most fragmented and degraded ecosystems (Davies et al., 2010; Kingsford, 2000). The focus of most barrier mitigation actions in the MDB to date has been on restoring passage across larger dams along the main river channel (Barrett & Mallen‐Cooper, 2006). Although some fishways have been designed to facilitate movement of smaller fish, they have predominantly targeted large‐bodied, highly mobile species (Baumgartner et al., 2014). Furthermore, the spatial scale of dispersal for many small‐bodied MDB fishes often restricts their movements to headwater streams and wetlands away from the main channel (Harris et al., 2017). Habitat loss and fragmentation associated with the thousands of smaller barriers in headwater streams have therefore likely contributed to the widespread decline of many smaller and more sedentary MDB fishes, including *N. australis* (Brauer et al., 2018; Cole et al., 2016; Hammer et al., 2013; Huey et al., 2017). It is perhaps surprising then, that there have been relatively few studies explicitly testing the genetic effects of anthropogenic fragmentation on small‐bodied fishes in the MDB. One recent example in the neighbouring Yarra River catchment, however, combined a large empirical data set with spatially explicit simulations to examine the role of artificial barriers in driving local‐scale patterns of genetic variation for river blackfish (*Gadopsis marmoratus*), a small and sedentary species also found in the MDB (Coleman et al., 2018). Based on eight microsatellite loci, genetic diversity was found to be lower for populations above barriers in small streams, with several isolated populations also exhibiting signs of inbreeding. In addition, their simulations demonstrated that power to detect recent impacts of barriers could be improved by increasing the number of loci used, highlighting the benefit of modern genomic data for conservation genetics.

An unprecedented severe and prolonged drought between 1997 and 2010 caused catastrophic loss of habitat and local extirpation for some *N. australis* populations, particularly in the lower Murray (Hammer et al., 2013; Wedderburn et al., 2012). In response, an emergency conservation breeding and restoration programme was implemented in the lower MDB (Attard et al., 2016; Hammer et al., 2013), and additional breeding and translocations among several headwater populations have been initiated (D. Gilligan and P. Rose, *personal communication*). As the impacts of climate change intensify, proactive conservation management interventions, such as those already underway for *N. australis*, will be increasingly considered for other species inhabiting the MDB and fragmented freshwater ecosystems elsewhere in the world. Indeed, a recent study incorporating physiological and functional traits with species distribution models for 23 fish species predicted severe declines in taxonomic and functional diversity of MDB fish communities in the coming decades due to climate change (de Oliveira et al., 2019). Managing regulated river systems to provide environmental flows, habitat restoration and other measures to re‐establish connectivity among habitat patches (e.g., installation of fishways) have the potential to address some impacts and should continue to be priorities for conservation and water management. Nonetheless, these long‐term, landscape‐scale measures are often constrained by competing interests related to political and socio‐economic issues (Davis et al., 2015).

Additionally, many species may be already depleted to the point where improved environmental conditions alone will not be sufficient to facilitate recovery. In this case, genetic rescue offers a potential solution for a broad range of threatened taxa (Ralls et al., 2018; Whiteley et al., 2015). However, despite strong evidence supporting the benefits of genetic rescue for fragmented populations, conservation managers are often reluctant to adopt these measures (Frankham, 2015). We suggest that the impacts of recent habitat fragmentation may have been underappreciated for many species, and that estimates of population structure solely attributed to historical evolutionary processes have potentially led to management frameworks that actually reinforce fragmentation and isolation at the expense of species‐level genetic variation (sensu Coleman et al., 2013). There is also increasing evidence that natural selection can influence the evolutionary trajectory of small and fragmented populations (Brauer et al., 2017; Fraser, 2017; Wood et al., 2016). Critically for conservation, this indicates that adaptive divergence of small populations can occur quickly following fragmentation (Brauer et al., 2017) and that even very recently isolated populations may harbour novel adaptive diversity. It is therefore important to build evolutionary resilience by facilitating genetic exchange among isolated populations to restore natural evolutionary processes and maintain species‐level genetic variation, potentially valuable under a range of future selection regimes (Webster et al., 2017; Weeks et al., 2016).

There is a global biodiversity crisis unfolding in freshwater ecosystems with aquatic vertebrate populations declining by 80% over the last 50 years (Darwall et al., 2018). Restoring functional connectivity for aquatic communities across river basins via traditional mitigation approaches is simply not feasible within the time frame required to enable many currently threatened species to persist. There is also now strong empirical evidence that several long‐established beliefs central to prevailing conservation practices are overly cautious, and that the current local‐is‐best approach increases the prospect of managing species to extinction (Frankham et al., 2017; Pavlova et al., 2017; Weeks et al., 2016). Given widespread fragmentation, habitat loss and the ongoing global decline of freshwater biodiversity, a rapid paradigm shift is needed to empower conservation practitioners to take action before demographic issues become critical. There are risks associated with any proactive management intervention such as translocation or genetic rescue. These risks, however, need to be weighed against the ever‐increasing risk of doing nothing.

## CONFLICT OF INTEREST

None declared.

## Supporting information

Supplementary Material

## Data Availability

Reference sequences, SNP genotypes, sample coordinates and environmental data used in analyses are available on Dryad: https://doi.org/10.5061/dryad.3dp50.
